# Supplementary material for: The Regulation of the Growth and Pathogenicity of Valsa mali by the Carbon Metabolism Repressor CreA
Source: Int J Mol Sci. 2023 May 25;24(11):9252. doi: 10.3390/ijms24119252 (PMC10253104; doi:10.3390/ijms24119252)
Supplement: Supplementary file 1 [file ijms-24-09252-s001.zip › ijms-2309232-supplementary.pdf]

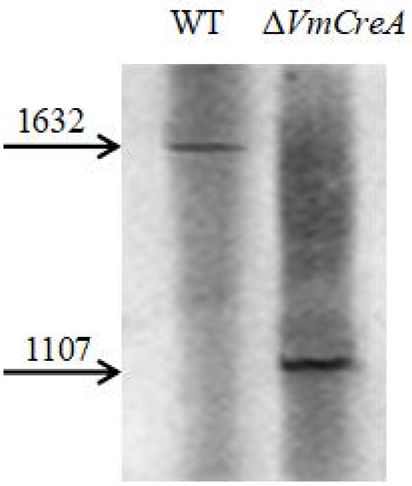

Figure S1. Southern blotting result of WT and ***ΔVmCreA***

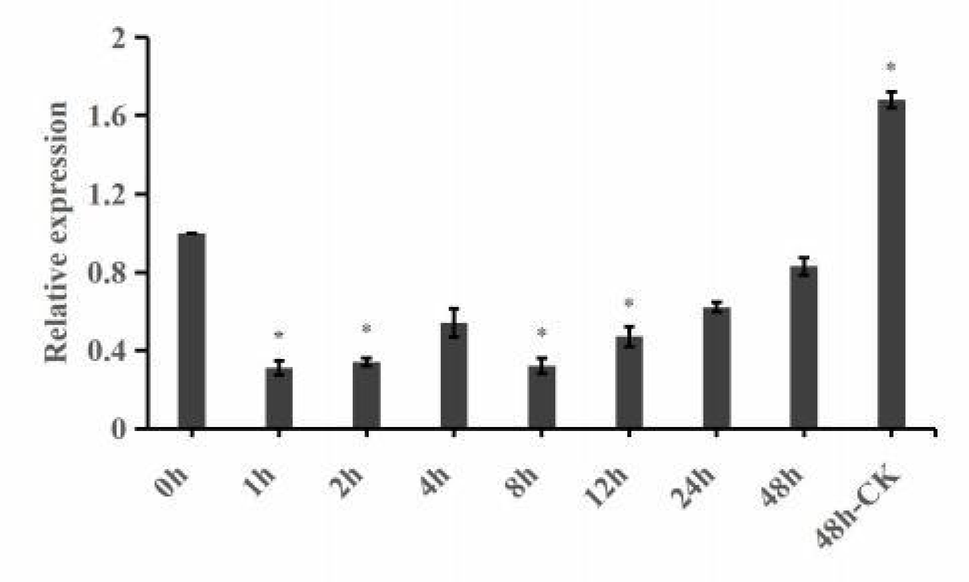

Figure S2. Analysis of ***VmCreA*** **relative expression level induced by glucose in different phase**. The single asterisk indicate significant differences at $p < 0.05$.

Table S1 Primers used in this study

Primer name	Primer sequence (5' to 3')
VmCreA-F	AAGTAGATAACGCTGCTCG
VmCreA-R	CCTTCACTACGGTAAACCC
VmCreA-U-F	AACCAGTCCGTGACCAGA
VmCreA-U-R	ATTCATTGTTGACCTCCACTTCATACCTCCATCCACCTG
VmCreA-D-F	GGGCAAAGGAATAGAGTAGACTGGCGACCACGGAACATA
VmCreA-D-R	AAACCAACCAGCCGTCAA
HPH-F	AGTGGAGGTCAACAATGAAT
HPH-R	TCTACTCTATTCCTTTGCCC

VmCreA-YZ-F1	TCGGGAAAGCCTCGGTAA
VmCreA-YZ-R1	CAAAGTGCCGATAAACATAAC
VmCreA-YZ-F2	TGGAGCGAGGCGATGTTC
VmCreA-YZ-R2	CGCATCTCGGCATTGGAC
HPH-YZ-F	TATTAGCAGACAGGAACGAGGAC
HPH-YZ-R	CTTCTGCGGGCGATTTGTGTA
VmCreA-HB-F	ACTCACTATAGGGCGAATTGGGTACTCAAATTGGTTAGGCGTGAGATACAGAAGG
VmCreA-HB-R	CACCACCCCGGTGAACAGCTCCTCGCCCTTGCTCACGGGTTTACCGTAGTGAAGG
eGFP-F	ATGGTGAGCAAGGGCGAGGA
eGFP-R	ACTAGTTTACTTGTACAGCTCGTCCATGC
GAPDH-F	TCAGAACAAGTTCGAGGGCGACAA
GAPDH-R	TGAGGGCAATAGAGGGCTTGTTCA
VM1G_02565-F	GCAACGGAGGCACGAAGA
VM1G_02565-R	TTGTAAACGGTAGCGGAAGAG
VM1G_02197-F	GGCATCCTCGCCGTAGCA
VM1G_02197-R	CCTTCAAGCCCGGTGCAAAC
VM1G_03115-F	GGAAACCGAAGCGTCTACAA
VM1G_03115-R	TCATCCTTAGCCAGGTTTGTG
VM1G_03512-F	TGCCAGCACGGCGGTTAG
VM1G_03512-R	GACTCCTCTATCACTGCGGGTA
VM1G_04322-F	CGCCACCATCACAACCCTC
VM1G_04322-R	TCGCTTGGCTTGTATGGGTG
VM1G_05544-F	AAATGGGTCGCCGAAAGTAAGG
VM1G_05544-R	AATGTCGCCGTCCAGGGTGT
VM1G_04032-F	GATGGTTTCCACGCTCTTCG
VM1G_04032-R	TGTGGCGTGGGCGTGGTT
VM1G_06261-F	GTCGCAGGTCGCAGAGTATGTC
VM1G_06261-R	CACCCTGGCTGGAACTGACC
VM1G_08966-F	CAGTTGGCAGGATACCGTGAG
VM1G_08966-R	GGGTGCCGAAGCCGTAGA
VM1G_08033-F	GCACGCCGTTCTGGCTGTTG
VM1G_08033-R	CGACTTCGTCACGCTGTCCTCC
VM1G_07516-F	GCTGGGAAGGAACTTTGGC
VM1G_07516-R	CTCGGTGTCGAAGGTGATGGTC
VM1G_10963-F	GGTGGTTCCCTCGGTGTTCC
VM1G_10963-R	CAAGGGAGTTACTGGAGGTGGC
VM1G_10438-F	CGTAGTAGCCCTGCTCCGTGTT
VM1G_10438-R	CCGCCGACGACGAGCACA
